# Professional Exposure to Goats Increases the Risk of Pneumonic-Type Lung Adenocarcinoma: Results of the IFCT-0504-Epidemio Study

**DOI:** 10.1371/journal.pone.0037889

**Published:** 2012-05-24

**Authors:** Delphine Lutringer-Magnin, Nicolas Girard, Jacques Cadranel, Caroline Leroux, Elisabeth Quoix, Vincent Cottin, Corinne Del Signore, Marie-Paule Lebitasy, Geneviève Cordier, Philippe Vanhems, Jean-François Mornex

**Affiliations:** 1 Hospices Civils de Lyon, Lyon, France; 2 Université de Lyon, Lyon, France; 3 Université Claude Bernard Lyon 1, Lyon, France; 4 UMR 5558 CNRS, Laboratoire de biométrie et biologie évolutive, Lyon, France; 5 UMR754 INRA, Rétrovirus et pathologie comparée, Lyon, France; 6 Service de Pneumologie, Hôpital Tenon, Paris, France; 7 Service de pneumologie, Hôpital Hautepierre, Strasbourg, France; 8 Intergroupe Français de Cancérologie Thoracique, Paris, France; Dartmouth College, United States of America

## Abstract

Pneumonic-type lung adenocarcinoma (P-ADC) represents a distinct subset of lung cancer with specific clinical, radiological, and pathological features. Given the weak association with tobacco-smoking and the striking similarities with jaagsiekte sheep retrovirus (JSRV)-induced ovine pulmonary adenocarcinoma, it has been suggested that a zoonotic viral agent infecting pulmonary cells may predispose to P-ADC in humans. Our objective was to explore whether exposure to domestic small ruminants may represent a risk factor for P-ADC. We performed a multicenter case-control study recruiting patients with P-ADC as cases and patients with non-P-ADC non-small cell lung cancer as controls. A dedicated 356-item questionnaire was built to evaluate exposure to livestock. A total of 44 cases and 132 controls were included. At multivariate analysis, P-ADC was significantly more associated with female gender (Odds-ratio (OR) = 3.23, 95% confidence interval (CI): 1.32–7.87, *p* = 0.010), never- smoker status (OR = 3.57, 95% CI: 1.27–10.00, *p* = 0.015), personal history of extra-thoracic cancer before P-ADC diagnosis (OR = 3.43, 95% CI: 1.10–10.72, *p* = 0.034), and professional exposure to goats (OR = 5.09, 95% CI: 1.05–24.69, *p* = 0.043), as compared to other subtypes of lung cancer. This case-control suggests a link between professional exposure to goats and P-ADC, and prompts for further epidemiological evaluation of potential environmental risk factors for P-ADC.

## Introduction

Lung cancer is the leading cause of cancer-related death worldwide, and is related to cigarette smoking in the majority of patients. However, the disease also arises in individuals who are never or little smokers, then mostly presenting with adenocarcinoma-type histology [1], [2]. Among these tumours, pneumonic-type adenocarcinoma (P-ADC) represents a subset with a distinct pathological and radiological presentation and a specific clinical course: higher female: male ratio, lower relationship with tobacco smoking, predominant lepidic growth, diffuse alveolar consolidation [3], [4]. The carcinogenesis of this tumour entity is poorly understood.

Ovine pulmonary adenocarcinoma is a naturally occurring lung cancer that arises in sheep infected by jaagsiekte sheep retrovirus (JSRV) [5]–[7]. It has been described for a long time as an animal model for human P-ADC, as the two tumours share strikingly similar clinical, radiological and pathological features [7], [8]. Animals with ovine pulmonary adenocarcinoma present with progressive dyspnea, abundant bronchorrhea, cough, anorexia and cachexia. As in its human counterpart, lesions present as multiple disseminated nodules and consolidation of variable size, with a morphologic pattern of adenocarcinoma with predominant lepidic growth. Both diseases originate from alveolar type II and Clara cells, although P-ADC may also contain bronchiolar mucoid cells [6].

A viral etiology of P-ADC has been suggested for years. Results are still conflicting [9], [10], but no direct biological evidence of a specific role for any retrovirus has been reported in human P-ADC so far. In this study, we approached this question from an epidemiological point of view. We hypothesized that, given the absence of - or the lower - direct exposure to known carcinogens, patients with P-ADC may represent a subgroup for which a viral agent, possibly related to JSRV and infecting pulmonary cells, may predispose to cancer. As previous attempts to detect viruses from human P-ADC have been largely unsuccessful, we undertook a case-control approach to determine whether exposure to domestic small ruminants might represent a risk factor for P-ADC in humans.

## Methods

### Study participants

This study was conducted as an ancillary study of two prospective phase II trials evaluating as first-line treatment of P-ADC the efficacy of gefitinib (IFCT-0401; clinicaltrialsID: NCT00198380) and the comparison of two strategies erlotinib *vs.* carboplatine paclitaxel (IFCT-0504; clinicaltrialsID: NCT00384826) [11].

The present case-control study aimed at determining whether chronic exposure to domestic small ruminants may increase the risk factor of P-ADC compared to other lung cancers. A case-control design was used given the low incidence of the disease. Participants were prospectively recruited in 11 regional university hospitals from January 2004 to March 2008. All centers used the same study design. Cases were consecutive patients, who consulted during the study period for prevalent or incident P-ADC, as defined based upon radiological-pathological criteria [3], including 1) histologically or cytologically-proven lung adenocarcinoma with predominant lepidic growth, *i.e.* bronchioloalveolar carcinoma or adenocarcinoma with bronchioloalveolar features according to the 2004 World Health Organisation classification [12], 2) multiple bilateral consolidations possibly showing air bronchogram at chest computed tomography, in the absence of pulmonary infection, 3) no endobronchial macroscopic lesion, and 4) no extra-thoracic metastases. Exclusion criteria were 1) lobar or complete lung atelectasis, and 2) personal history of extra-thoracic cancer in the 5 years prior to P-ADC diagnosis.

Three controls, i.e. one patient with non-P-ADC lung adenocarcinoma and two patients with non-adenocarcinoma-type non-small cell lung cancer, were recruited for each case. This design was chosen to address potential bias related to common risk factors both for lung adenocarcinoma and furthermore lung cancer, including tobacco smoking. Controls had to be recruited within a 6-month period in the same hospital department as cases and were not matched with cases regarding gender, age, or tobacco-smoking history.

All patients provided written informed consent before enrollment. The regional ethics committee of the Hospices Civils de Lyon and local institutional review boards of participating institutions approved the study.

### Data collection

Data were collected using a dedicated 356-item questionnaire (available in french upon request) that was administrated by the physician in charge of the patient during a face-to-face interview. A written protocol was set up to ensure standardization in the procedures to collect data. The questionnaire addressed the frequency, the type (professional or leisure), and the age-period (0–12, 13–20, 21–40 and >40 years-old) of contacts, and the domestic animals (family and species) the patient was exposed to: (1) *Bovidae* that include sheep (*Ovis aries*), goats (*Capra hircus*), and cattle (*Bos taurus*), (2) *Equidae* especially horses (*Equus caballus*), donkeys (*Equus asinus*), (3) *Suidae* with pigs (*Sus scrofa*), (4) *Leporidae* with rabbits (*Oryctolagus cuniculus*) (5) *Phasianidae*, with domestic chickens (*Gallus gallus*) and turkeys (*Meleagris gallopavo*), and (6) *Anatidaed*, especially ducks (*Anas platyrhynchos*) and geese (*Anser domesticus*). If significant contact was identified, more detailed data were collected using a pre-established list of closed questions: What was the patient occupation? How many animals were in the farm? Did the farm experience epidemic infection? Did the patient personally participate in the feeding/milking/care/slaughtering processes? Was the patient exposed to animal blood, viscera, or fetal tissue? Collected data also included demographics, county of residency and frequency of country stay, detailed smoking history, including second-hand tobacco exposure, alcohol consumption, job history, environmental exposures, and complete personal and familial medical history, especially of cancer and pulmonary non-neoplastic disease. Never smokers were defined as individuals who smoked less than 100 cigarettes in a lifetime. Little smokers were individuals who had a tobacco-consumption history lower than 15 pack-years.

### Statistical methods

Categorical variables were compared using the Chi-2 test and continuous variables using the Student's test. Multivariate logistic regression was used to determine variables independently associated with P-ADC *vs.* other lung cancers (entry *p*<0.15). Results were considered significant at the 0.05 levels. Independent variables were selected using a backward selection technique based on the likelihood ratio test, using the SPSS software program (Chicago, IL), version 17. Preliminary data suggested that about 30% of French individuals >40 years old have been exposed to domestic small ruminants. A group-sequential method, based on a triangular test, was used to calculate the number of patients to include. We estimated that 42 cases and 126 controls would allow 85% power to detect a 6-fold increase in the risk of P-ADC in patients exposed to domestic small ruminants.

## Results

### Study population

A total of 44 patients with P-ADC (cases) were interviewed and completed the questionnaire. We were able to recruit 132 control patients, including 44 patients with non-P-ADC lung adenocarcinoma and 88 patients with non-adenocarcinoma-type non-small cell lung cancer. Population characteristics are shown in Table 1. As expected in France, the majority of patients were Caucasian (88% of cases and 94% of controls; *p* = 0.310). As required in the study protocol for each individual case, all controls were recruited at the same medical center.

**Table 1:** Population characteristics.

	Casesn = 44 (%)	Controlsn = 132 (%)	*P*
**Demographic data**			
**Gender**			
Male	20 (45)	103 (78)	<0.001
Female	24 (55)	29 (22)	
**Smoking history**			
Smoker	27 (61)	121 (92)	0.001
Never-smoker	17(39)	11 (8)	
**Pack-years**			
0	17 (39)	12 (9)	<0.001
1–25	15 (34)	28 (21)	
26–50	9 (20)	46 (35)	
>50	3 (7)	46 (35)	
**Personal and family history**			
**Lung diseases**			
No	29 (76)	86 (65)	0.928
Yes	15 (24)	46 (35)	
**X-Ray exposure**			
No	41 (93)	128 (97)	0.265
Yes	3 (7)	4 (3)	
**Personal history of cancer**			
No	36 (82)	122 (92)	0.080
Yes	8 (18)	10 (8)	
**Family history of lung cancer**			
No	42 (95)	115 (87)	0.123
Yes	2 (5)	17 (13)	

Case (P-ADC) and control (non-P-ADC) groups were compared using univariate analysis with the Chi-2 test.

Univariate analysis showed that cases were more frequently women (*p*<0.001), and never- or little-smokers than controls (*p* = 0.001 and *p*<0.001, respectively). There was no significant difference between cases and controls regarding mean age at tumour onset (64 and 60 years-old, respectively; *p* = 0.112), frequency and type of concurrent non-neoplastic lung disease (*p* = 0.928), X-ray chronic exposure (*p* = 0.265), personal history of cancer more than 5 years before P-ADC diagnosis (*p* = 0.080), or family history of lung cancer (*p* = 0.123).

### Exposure to livestock

To ensure an optimal coverage of activities linked with exposure to livestock, we collected data about the frequency, the age period, the leisure and/or professional setting, and the type of animals the patients with lung cancer had contacts with (Table 2). Overall, 34% of individuals of the cohort had contact with domestic small ruminants. Univariate analysis did not identify any significant differences in leisure-related variables among cases and controls (Table 2); of note, exposure during childhood (either 0–12 years or 13–20 years) was not different between the two groups.

**Table 2:** Exposure to livestock in the study population.

	Casesn = 44 (%)	Controlsn = 132 (%)	*P*
**Leisure exposure**			
**Animals**			
Sheep	9 (20.5)	31 (23.5)	0.644
Goats	19 (43.2)	47 (35.6)	0.369
Poultry	25 (56.8)	70 (53.0)	0.662
Cows	16 (36.4)	53 (40.2)	0.631
Pigs	12 (27.3)	37 (28.0)	0.923
Horses	11 (25.0)	25 (18.9)	0.394
Rabbits	23 (52.3)	65 (49.2)	0.728
**Frequency/Age (≥1 country stay per week)**			
0–12 years-old	15 (34.1)	57 (43.2)	0.288
12–20 years-old	13 (29.5)	53 (40.2)	0.208
20–40 years-old	12 (27.3)	33 (25.0)	0.765
>40 years-old	9 (20.5)	35 (26.5)	0.421
**≥1 family member farmer, shepherd or breeder**	19 (43.2)	52 (39.4)	0.657
**Professional exposure**			
**Occupation**			
Farmer	6 (13.6)	33 (25.0)	0.352
Breeder	3 (6.8)	15 (11.4)	0.567
Shepherd	0	4 (3.0)	0.573
Butcher	2 (4.5)	1 (0.8)	0.155
Laboratory worker	0	1 (0.8)	1
**Type of animals**			
Sheep	3 (6.8)	9 (6.8)	1
Goats	4 (9.1)	4 (3.0)	0.095
Poultry	4 (9.1)	19 (14.4)	0.366
Cows	3 (6.8)	21 (15.9)	0.128
Pigs	1 (2.3)	13 (9.8)	0.194
Horses	5 (11.4)	9 (6.8)	0.343
Rabbits	0 (0)	0 (0)	-

Case (P-ADC) and control (non-P-ADC) groups were compared using univariate analysis with the Chi-2 test.

Conversely, even if the type of occupation was not identified as a significant variable, exposure to goats tended to be more frequent in cases *vs.* controls (9% *vs.* 3%, respectively; *p* = 0.095). Using all the informations collected, we then looked into details of the professional activities; despite the potential limitation of limited size of the groups, there was a trend for cases to be more frequently involved in the processes of goat feeding (9% *vs.* 3% in controls; *p* = 0.112), care (9% *vs.* 2%; *p* = 0.032), and litter (7% vs. 2%; *p* = 0.173) processes. No significant difference in the age of exposed cases and controls was identified. However, living in the same household as an individual working with domestic small ruminants did not increase the risk of P-ADC (*p* = 0.657).

### Association analyses

The following variables identified at univariate analysis to potentially be associated with P-ADC when compared to non-P-ADC were included in the multivariate analysis model: age, gender, smoking status and pack-years, personal history of extra-thoracic cancer more than 5 year before P-ADC, and professional exposure to goats.

Overall, the analysis confirmed that P-ADC was significantly more associated with female gender (Odds-ratio (OR) = 3.23, 95% confidence interval (CI): 1.32–7.87, *p* = 0.010), never- smoker status (OR = 3.57, 95% CI: 1.27–10.00, *p* = 0.015), personal history of extra-thoracic cancer (OR = 3.43, 95% CI: 1.10–10.72, *p* = 0.034), and professional exposure to goats (OR = 5.09, 95% CI: 1.05–24.69, *p* = 0.043), as compared to non-P-ADC lung cancer (Table 3).

**Table 3:** Factors independently associated with pneumonic-type lung adenocarcinoma.

	Casesn = 44 (%)	Controlsn = 132 (%)	Odds-Ratio	[CI 95%]	*P*
**Gender**					
Male	20 (45)	103 (78)	1		
Female	24 (55)	29 (22)	3.23	1.32–7.87	0.010
**Smoking status**					
Smoker	27 (61)	121 (92)	1		
Never smoker	17(39)	11 (8)	3.57	1.27–10	0.015
**Personal history of cancer**					
No	36 (82)	122 (92)	1		
Yes	8 (18)	10 (8)	3.43	1.10–10.72	0.034
**Professional exposure to goats**					
No	40 (81)	128 (97)			
Yes	4 (9)	4 (3)	5.09	1.05–24.69	0.043

Multivariate analysis was performed using log regression model including significant variables at univariate analysis with *p*<0.15.

CI: Confidence Interval.

## Discussion

The identification of environmental risk factors that may predispose to lung cancer requires a deep analysis of leisure and professional activities in specific subgroups of patients for whom tobacco smoking may not represent a major carcinogen. In this study, we show that case-control study is an appropriate approach to assess potential associations between P-ADC and exposure to domestic small ruminants, as previous virology studies have failed to isolate a causal infectious agent.

We were able to recruit the planned number of cases and controls in a reasonable timeframe for a rare disease. Moreover, using this approach, our results suggest that, among non-small lung cancers, P-ADC may occur more frequently in patients exposed to goats in an occupational setting, with an OR of 5.1 that is significantly higher than those previously reported for most of the non-tobacco-related risk factors for lung cancer such as 1.3 to 1.4 for diesel exhaust exposure, 1.1 to 1.3 for coffee consumption, and 1.3 to 1.7 for silica exposure [13]–[15].

P-ADC is a rare tumour, which corresponds to a specific clinical-radiological-pathological entity within lung adenocarcinomas [3], [4]. From a pathological perspective, P-ADC is frequently classified as diffuse mucinous bronchioloalveolar carcinoma [3], [16], [17]. Higher female: male ratio, higher number of never- or little-smokers, and higher frequency of previous cancer were associated with an increased risk of P-ADC as compared to other subtypes of lung cancer, as previously reported [3], [16]–[22]. The latest findings suggest that genetic susceptibility plays a significant role in the carcinogenesis of P-ADC, as highlighted by recent genome-wide association studies conducted in never-smokers [23]. The clinical characteristics of our cohort of cases were thus very similar to that of previously reported series of patients with P-ADC, therefore suggesting our findings regarding exposure to domestic small ruminants to be relevant.

Some limitations of our analysis must be addressed. First, as in any case-control studies, a memory bias could have occurred for the cases. In our study, the fact that controls, as well as cases, were sick patients and thus had to do a similar memory effort to remember past exposures may have partially controlled this bias. Secondly, we identified a risk factor between patients with different types of lung cancers, namely P-ADC *vs.* other lung carcinomas, but not *vs.* healthy controls. The statistical power of our analysis would be improved with a larger sample size, facilitating identification of additional interactions and comparison to other risk factors previously identified. However, feasibility may be limited by the rarity of P-ADC. Finally, *Epidermal Growth Factor Receptor (EGFR)* mutations, which are major oncogenic drivers in lung carcinoma tumours exhibiting predominant lepidic growth [24], were not assessed, as collection of biological materials was not included in the design of the 2 associated trials, which were designed before these mutations were discovered.

The rationale supporting a potential association between P-ADC and exposure to goats is not fully understood. Besides a potential exposure of farmers to chemical carcinogens found in artificial fertilizers –that was not recorded in our study-, our baseline hypothesis was that morphological similarities between JSRV-related ovine pulmonary carcinoma and human P-ADC suggest a role for a zoonotic agent, such as JSRV or JSRV-related virus infection playing a role in human cancer induction [7], [8]. Identifying a more frequent exposure to sheep in patients with P-ADC would have further supported this hypothesis. An association with goat exposure was not anticipated. However, our findings prompt further research studies towards caprine specific infectious agents, including *enzootic nasal tumor virus* (ENTV), a beta retrovirus closely related to JSRV and responsible for an ethmoid tumour in goats associated with the transformation of nasal epithelial cells [25]. JSRV and ENTV are two closely related viruses having many biological features in common. Further supporting this hypothesis, meat workers have already been reported to harbor a higher risk of cancer [26].

Hyaluronidase type 2 (HYAL2) is the ubiquitous receptor for both the JSRV and ENTV envelope glycoproteins. HYAL2 is expressed at the cell surface and mediates infection by retroviral vectors pseudotyped with the JSRV or ENTV envelope proteins [27]–[29]. Several studies have explored the possible link between retroviruses of the JSRV family and human lung adenocarcinoma [9], [27], [30]–[33]. An immunohistochemistry study suggested that a JSRV-related capsid protein might be expressed in about 30% of human bronchioloalveolar carcinomas, and 26% of lung adenocarcinoma tumours [30]. Endogenous JSRV DNA sequences, integrated counterparts of the exogenous JSRV, have been detected in bronchioloalveolar tumor tissues from Sardinian patients [31], as well as in the blood from African patients without lung tumours [32]. Other studies using more specific PCR-based analyses failed to detect any exogenous or endogenous JSRV or beta retrovirus related sequences in human adenocarcinoma [9], [33]. These data discussing a role for JSRV in human lung cancer have yet to be further validated. In this respect, this first epidemiological study gives encouraging signal to continue to assess a viral component in P-ADC. Of note, a recent report suggested that *Human T-cell Lymphotropic Virus type I*, a member of the retrovirus family related to JSRV and ENTV, may represent a risk factor for bronchioloalveolar carcinoma [10].

To conclude, this study suggests an independent link between goat exposure and P-ADC. As the biological mechanisms supporting such association remain unknown, these results should be considered as hypothesis-generating, and prompt for further epidemiological studies especially in a more selected population: never- or little-smokers, living in the countryside, possibly in JSRV/ENTV endemic regions, and working with domestic small ruminants. Better quantification of animal exposure is also needed, similarly to earlier studies conducted with carcinogens [34]. Meanwhile, environmental questionnaire might be included in clinical studies enrolling patients with P-ADC.
